# Correction: Triethylamine inhibits influenza A virus infection and growth via mechanisms independent of viral neuraminidase and RNA-dependent RNA polymerase

**DOI:** 10.1371/journal.pone.0349522

**Published:** 2026-05-20

**Authors:** Masaki Shoji, Kensuke Nakaoka, Momiji Ishikawa, Yusuke Kasai, Tomoyuki Esumi, Etsuhisa Takahashi, Hiroshi Kido, Hiroshi Imagawa, Takashi Kuzuhara

The eighth author’s name is spelled incorrectly. The correct name is: Hiroshi Imagawa.
