# Supplementary material for: Triethylamine inhibits influenza A virus infection and growth via mechanisms independent of viral neuraminidase and RNA-dependent RNA polymerase
Source: PLoS One. 2025 Aug 7;20(8):e0329964. doi: 10.1371/journal.pone.0329964 (PMC12331046; doi:10.1371/journal.pone.0329964)
Supplement: S1 Data — (PDF) [file pone.0329964.s002.pdf]

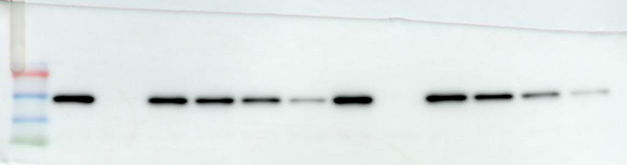

WB image_PR8_influenza NP

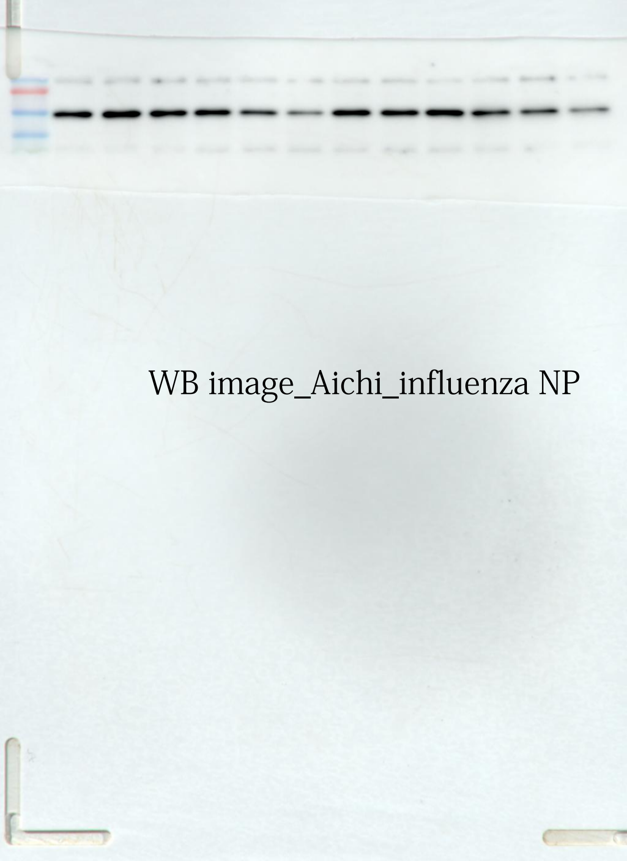

WB image_Aichi_influenza NP

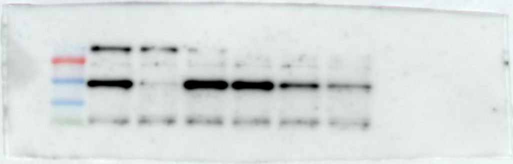

WB image_WSN_influenza NP

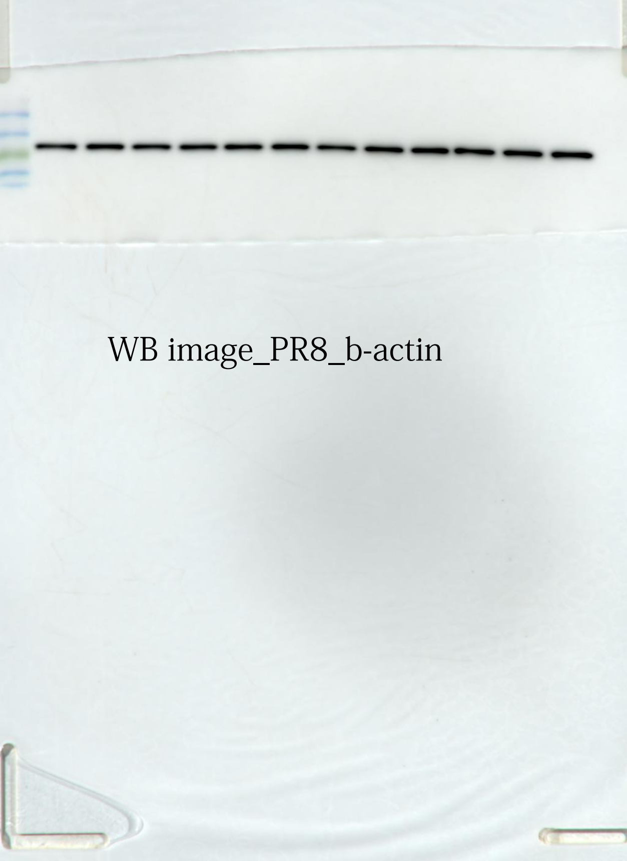

WB image_PR8_b-actin

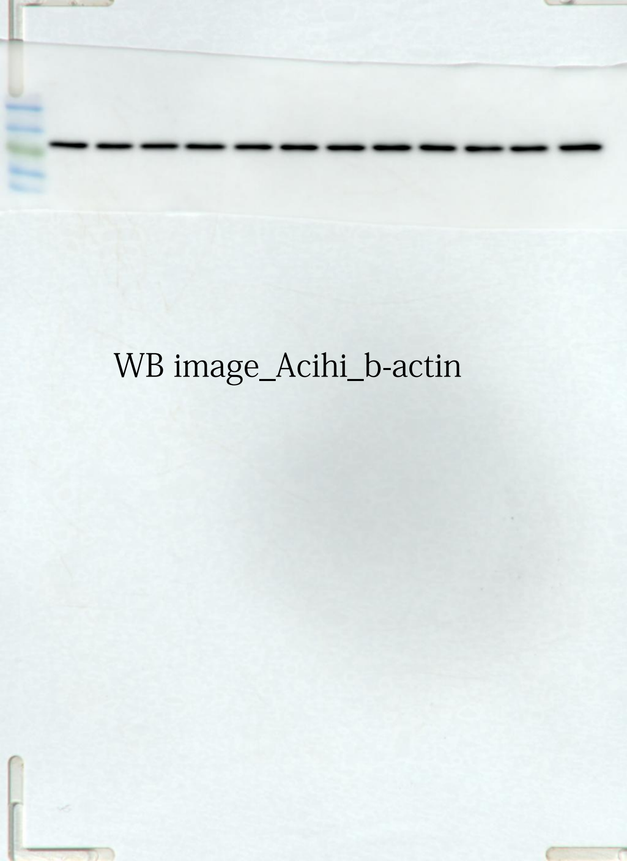

WB image_Acihi_b-actin

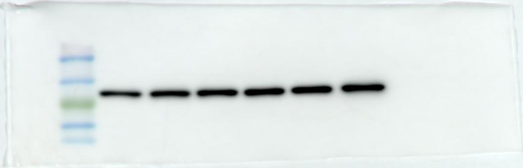

WB image_WSN_b-actin
