# Supplementary material for: Triethylamine inhibits influenza A virus infection and growth via mechanisms independent of viral neuraminidase and RNA-dependent RNA polymerase
Source: PLoS One. 2025 Aug 7;20(8):e0329964. doi: 10.1371/journal.pone.0329964 (PMC12331046; doi:10.1371/journal.pone.0329964)
Supplement: Attachment — Submitted filename: Response to reviewers comments.docx [file pone.0329964.s004.docx]

**Responses to Reviewers’ comments**

Manuscript Number: PONE-D-25-29309

Masaki Shoji, Kensuke Nakaoka, Momiji Ishikawa, Yusuke Kasai, Tomoyuki Esumi, Etsuhisa Takahashi, Hiroshi Kido, Hiroshi Imawaga, Takashi Kuzuhara

“**Triethylamine inhibits influenza A virus infection and growth via mechanisms independent of viral neuraminidase and RNA-dependent RNA polymerase**”

Dear Professor James Guevara Pulido,

We would like to thank you and the reviewers for their positive comments on our manuscript and their constructive suggestions for improvement. As per the reviewers’ comments, we have added new experimental information and revised the manuscript accordingly.

Please find our point-by-point responses (in blue) to the comments of the reviewers (in ***italics***) below.

We believe that the manuscript has been revised appropriately as per the opinions expressed, and we hope that it is now suitable for publication in *PLOS ONE*.

Thank you for your consideration. I look forward to hearing from you.

Sincerely yours,

Takashi Kuzuhara, Ph.D.

Professor, Laboratory of Biochemistry, Faculty of Pharmaceutical Sciences,

Tokushima Bunri University

180 Nishihamahouji, Yamashirocho, Tokushima-city, 770-8514, Japan

Tel.: +81 (88) 602-8477, Fax: +81 (88) 655-3051, E-mail: [kuzuhara@ph.bunri-u.ac.jp](mailto:kuzuhara@ph.bunri-u.ac.jp)

**Editor’s comments**

**Additional Editor Comments:**

*The work is engaging but should include details about the research limitations in both the manuscript's body and conclusions. It would also be beneficial to describe the selectivity indices of triethyl amine in vitro and discuss how, as a small and highly reactive molecule, triethyl amine is likely to have negligible bioavailability in vivo. This addresses the authors' challenge in accounting for this discrepancy.*

**Response:**

We sincerely thank the editor for the constructive and insightful feedback. In response, we have made several revisions to address the points raised.

We have added a new "Research limitations" section, placed between the Discussion and Conclusion sections (see pages 15–16, lines 427–460). This section explicitly outlines the limitations of our current study. Notably, we have now discussed the lack of *in vivo* data, as well as concerns related to the bioavailability and pharmacokinetic properties of triethylamine (Et₃N).

Next, we have evaluated and described the selectivity index (SI) of Et₃N against influenza A virus infection, as shown in Table 1 of the Results section (see page 11, lines 300–314). The SI was calculated using the method described in the Materials and methods section (see page 6, lines 166–174), based on CC₅₀ values determined in MDCK cells by the WST-8 assay (Fig. 1B) and IC₅₀ values derived from immunofluorescence and Western blot analyses for A/PR/8/34, A/WSN/33, and A/Aichi/2/68 strains (Figs. 2 and 3A–C). The calculated SI values ranged from approximately 10 to over 50, indicating a moderate to high degree of selectivity *in vitro*.

Furthermore, in the newly added Research limitations section, we acknowledge that no *in vivo* assessments of Et₃N’s safety, toxicity, or pharmacokinetics have been conducted. We note that the observed IC₅₀ values of Et₃N ranged from approximately 178 to over 1,000 µM *in vitro*, which are relatively high and may not be achievable or safe at pharmacologically relevant concentrations *in vivo*. Åkesson et al. (Åkesson et al., 1989) reported that the plasma half-life of Et₃N in humans is nearly three hours, suggesting that its bioavailability is not negligible. We now emphasize that further chemical optimization would be essential to improve its drug-like properties.

Lastly, the Conclusion section has been revised accordingly to reflect these points and ensure alignment with the Research limitations section.

**Reviewer #1:**

**Comment 1:**

*-Why the authors did not study the effect of Et3N in vivo?*

**Response 1:**

We appreciate the reviewer’s insightful question regarding the lack of *in vivo* evaluation of Et₃N. In response, we have now included a detailed discussion in the newly added "Research limitations" section of the revised manuscript, and we have updated several other sections accordingly.

Although the selectivity index (SI) values of Et₃N against influenza A virus infection ranged from approximately 10 to over 50, its safety profile, toxicity, and pharmacokinetic properties have not yet been evaluated in animal models. As shown in Table 1, the *in vitro* IC₅₀ values of Et₃N ranged from approximately 178 to over 1,000 µM, which are relatively high and may not be achievable or safe at pharmacologically relevant concentrations *in vivo* settings. Moreover, due to its small molecular size and high reactivity, Et₃N is likely to exhibit low bioavailability *in vivo*. This limitation is now clearly acknowledged and discussed as a key challenge that must be addressed through further structural optimization.

To address this point comprehensively, we have incorporated relevant descriptions and data in the following sections of the revised manuscript:

**Abstract** (page 2, lines 38–39):
“Selectivity index values of Et₃N against influenza A virus infection, ranging from approximately 10 to over 50.”

**Materials and methods** (pages 6–7, lines 171–179):

We have added a subsection describing the method used for SI calculation:
“**The selectivity index (SI) calculation**...”
(Details of the formula, CC₅₀ and IC₅₀ values, and software used are provided.)

**Results** (page 11, lines 304–318):
We now describe how SI values were calculated from WST-8, IF staining, and WB results (Table 1). The SI values of Et₃N were:

From IF staining: 56 (A/PR/8/34), 36 (A/WSN/33), and 36 (A/Aichi/2/68)

From WB analysis: 15, 10, and 10, respectively
These values demonstrate moderate antiviral selectivity *in vitro*.

**Research limitations** (pages 15–16, lines 431–466):

We emphasize that all experiments were conducted solely in vitro using MDCK cells, and that further *in vivo* studies are essential for assessing the therapeutic potential of Et₃N. The physicochemical characteristics of Et₃N likely contribute to low systemic exposure *in vivo*, thereby limiting its translational feasibility.

**Conclusion** (pages 16–17, lines 468–479):

The conclusion section now reflects these limitations and suggests future directions. Specifically, we state that while Et₃N displays promising *in vitro* antiviral activity, its relatively high IC₅₀ values, and absence of *in vivo* data highlight the need for chemical optimization and further validation in physiologically relevant models.

**Comment 2:**

*-The rationale about thinking about testing ET3N against influenza virus is not clear.*

**Response 2:**

We thank the reviewer for this important comment regarding the rationale for investigating the anti-influenza activity of Et₃N. Et₃N is a small, basic organic compound commonly used as a proton (H⁺) acceptor in a wide range of industrial organic synthesis processes, including the manufacture of pharmaceuticals, agrochemicals, and polymers. While high concentrations of Et₃N are known to be toxic—particularly via inhalation, which can cause respiratory tract and lung damage—its potential biological and pharmacological effects at non-toxic concentrations have not been well explored, especially in the context of infectious diseases of the respiratory tract. Given the compound’s known reactivity and membrane-penetrating properties, we hypothesized that Et₃N may exert modulatory effects on virus–host interactions in epithelial cells. We thus speculated that Et₃N might influence influenza A virus infection and/or growth efficiency. Our study revealed that Et₃N enhances the survival of influenza A virus-infected MDCK cells and inhibits viral infection and growth *in vitro*, through mechanisms that appear to be independent of NA and RdRp functions. We have revised the Abstract and Introduction sections to clarify this rationale:

**Abstract** (page 2, lines 28–34):

“Triethylamine (Et_3_N) is a proton (H⁺) acceptor that is widely used in various industrial organic synthesis processes, including the production of pharmaceuticals, agrochemicals, and polymers. Inhalation of high Et_3_N concentrations can damage human respiratory tract and lungs. Given the compound’s known reactivity and membrane-penetrating properties, we hypothesized that non-toxic concentrations of Et₃N may exert modulatory effects on virus–host interactions in epithelial cells. We thus investigated the anti-influenza activity of Et_3_N and…”

**Introduction** (page 3, lines 74–76):

“Although Et_3_N has been investigated in various contexts, its biological and pharmacological effects of antiviral activity at non-toxic concentrations on infectious diseases of the respiratory tract have not been extensively studied. Given the compound’s known reactivity and membrane-penetrating properties, we hypothesized that Et₃N may exert modulatory effects on virus–host interactions in epithelial cells. We thus speculated that Et₃N might influence influenza A virus infection and/or growth efficiency.”

**Comment 3:**

*-Please check this phrase in the abstract "We investigated the antiinfluenza
activity of Et3N and found that it increased the viability of influenza A H1N1" does "increased the viability" is right"?*

**Response 3:**

We thank the reviewer for pointing out this phrasing issue. We agree that the original phrase "increased the viability of influenza A H1N1" was ambiguous and could be misinterpreted as implying enhanced viral viability. Our intended meaning was that Et₃N improved the viability of host cells infected with influenza A virus. To clarify this, we have revised the phrasing from "increased" to "enhanced" in both the Abstract and Results sections as follows:

**Abstract** (page 2, line 34):

“…and found that it enhanced the…”

**Results** (page 8, line 235):

“Et_3_N enhanced influenza A H1N1 and H3N2 virus-infected MDCK…”

**Reviewer #2:**

*Specific comments:*

*This study demonstrated that triethylamine (Et3N), a commonly used industrial compound, exerts antiviral activity against influenza A H1N1 and H3N2 viruses in MDCK cells. At non-toxic concentrations, Et3N suppressed viral infection, reduced viral gene expression, and inhibited virus release and/or replication, although it did not target neuraminidase or RNA polymerase directly. Even if the current study offers a significant contributions influenza A virus treatment, it has numerous limitations that should be taken into account, including the following implications:*

**Response:**

We thank Reviewer#2 for their thoughtful suggestions and insights, which have enriched the manuscript and produced a better and more balanced account of the research.

**Limitations:**
**Comment 1:**
*1. The study was conducted only in vitro using MDCK cells; no in vivo or clinical validation was performed*.

**Response 1:**

We appreciate the reviewer’s insightful question regarding the lack of *in vivo* evaluation of Et₃N. In response, we have now included a detailed discussion in the newly added "Research limitations" section of the revised manuscript, and we have updated several other sections accordingly.

Although the selectivity index (SI) values of Et₃N against influenza A virus infection ranged from approximately 10 to over 50, its safety profile, toxicity, and pharmacokinetic properties have not yet been evaluated in animal models. As shown in Table 1, the *in vitro* IC₅₀ values of Et₃N ranged from approximately 178 to over 1,000 µM, which are relatively high and may not be achievable or safe at pharmacologically relevant concentrations *in vivo* settings. This limitation is now clearly acknowledged and discussed as a key challenge that must be addressed through further structural optimization.

To address this point comprehensively, we have incorporated relevant descriptions and data in the following sections of the revised manuscript:

**Abstract** (page 2, lines 38–39):
“Selectivity index values of Et₃N against influenza A virus infection, ranging from approximately 10 to over 50.”

**Materials and methods** (pages 6–7, lines 171–179):

We have added a subsection describing the method used for SI calculation:
“**The selectivity index (SI) calculation**...”
(Details of the formula, CC₅₀ and IC₅₀ values, and software used are provided.)

**Results** (page 11, lines 304–318):
We now describe how SI values were calculated from WST-8, IF staining, and WB results (Table 1). The SI values of Et₃N were:

From IF staining: 56 (A/PR/8/34), 36 (A/WSN/33), and 35 (A/Aichi/2/68)

From WB analysis: 15, 10, and 10, respectively
These values demonstrate moderate antiviral selectivity *in vitro*.

**Research limitations** (pages 15–16, lines 431–466):

We emphasize that all experiments were conducted solely in vitro using MDCK cells, and that further *in vivo* studies are essential for assessing the therapeutic potential of Et₃N. The physicochemical characteristics of Et₃N likely contribute to low systemic exposure *in vivo*, thereby limiting its translational feasibility.

**Conclusion** (pages 16–17, lines 468–479):

The conclusion section now reflects these limitations and suggests future directions. Specifically, we state that while Et₃N displays promising *in vitro* antiviral activity, its relatively high IC₅₀ values, and absence of *in vivo* data highlight the need for chemical optimization and further validation in physiologically relevant models.

**Comment 2:**

*2. The exact molecular target of Et3N remains unidentified, limiting mechanistic insight.*

**Response 2:**

While our results demonstrate that Et₃N does not inhibit NA or RdRp activity, the precise molecular targets responsible for its antiviral effects—whether viral or host cellular components—remain unidentified. Given that Et₃N is a typical Lewis base and a strong proton (H⁺) acceptor, we hypothesize that it may interfere with endosomal acidification, a critical step required for viral uncoating during entry into host cells. By buffering intracellular pH, Et₃N may suppress the pH-dependent conformational changes necessary for viral fusion, thereby inhibiting infection and growth.

However, this remains speculative, and we fully acknowledge that additional mechanistic studies are necessary to determine whether Et₃N affects viral entry, intracellular trafficking, host signaling pathways, or membrane dynamics involved in the influenza virus life cycle. To address this point, we have added the following text to the Research limitations section of the revised manuscript:

**Research limitations** (pages 16, lines 452–456):

“The molecular mechanisms underlying the antiviral activity of Et₃N remain unclear. While Et₃N did not inhibit NA or RdRp activity, its precise targets—whether viral components or host cellular factors—are still unknown. Further mechanistic studies are necessary to determine whether Et₃N interferes with viral entry, intracellular trafficking, host signaling pathways, or membrane dynamics involved in viral replication and release.”

**Comment 3:**
*3. Potential cytotoxicity and safety in humans, especially at therapeutic doses, were not assessed.*

**Response 3:**

We thank the reviewer for this important comment regarding the cytotoxicity and safety profile of Et₃N, particularly in the context of potential human application. In response, we evaluated the SI values of Et₃N against influenza A virus infection using standard cytotoxicity and antiviral assays in MDCK cells. The SI was calculated as the ratio of the 50% cytotoxic concentration (CC₅₀) to the 50% inhibitory concentration (IC₅₀), according to the formula: SI = CC₅₀ / IC₅₀. The CC₅₀ was obtained from WST-8 assay results (Fig 1B), and IC₅₀ values were derived from both IF and WB analyses (Figs 2 and 3A–C). The SI values ranged from approximately 10 to over 50, depending on the viral strain and assay method. These values suggest a favorable *in vitro* therapeutic window; however, we fully acknowledge that these findings are limited to MDCK cells, which are non-human epithelial cells. To address the broader concern regarding human safety and relevance, we have added a discussion in the Research limitations section emphasizing the need for further validation in human-derived models, such as primary bronchial epithelial cells or lung organoids. These models would provide a more physiologically relevant assessment of Et₃N’s safety and antiviral efficacy. We have also included this limitation in the Conclusion section, noting that while Et₃N shows promise as a potential antiviral scaffold, its relatively high IC₅₀ values, lack of *in vivo* validation, and the absence of testing in human systems significantly limit its immediate therapeutic applicability. The following sections were revised to reflect these points:

**Abstract** (page 2, lines 38–39):

“Selectivity index values of Et₃N against influenza A virus infection, ranging from approximately 10 to over 50.”

**Materials and methods** (pages 6–7, lines 171–179):

A detailed description of SI calculation methodology was added.

**Results** (page 11, lines 304–318):

Specific SI values were reported for each strain and assay method in Table 1.

**Research limitations** (page 16, lines 457–460):

“The antiviral effects of Et₃N were demonstrated only in MDCK cells. The reproducibility of these findings in human-derived systems, such as primary bronchial epithelial cells or lung organoids, has not yet been validated. Such studies would provide more physiologically relevant insights into the potential therapeutic application of Et₃N in humans.’’

**Conclusion** (Page 16–17, lines 468–479):

Expanded discussion on the lack of *in vivo* and human-derived model validation, and the need for further optimization of Et₃N.

**Comment 4:**
*4. The broad-spectrum antiviral potential of Et3N (against other viruses) was not evaluated.*

**Response 4:**

We thank the reviewer for this thoughtful comment. We acknowledge that while our study provides compelling *in vitro* evidence for the antiviral activity of Et₃N against influenza A virus, we did not evaluate its efficacy against other respiratory viruses. As such, the broad-spectrum antiviral potential of Et₃N remains to be determined. To address this limitation, we have now added a statement in the Research limitations section, emphasizing the importance of future studies that examine Et₃N’s antiviral activity against a wider range of respiratory pathogens, such as respiratory syncytial virus, coronaviruses (including SARS-CoV-2), rhinoviruses, adenoviruses, and parainfluenza viruses. We also revised the Conclusion section to reflect this point, noting that the lack of evaluation against other viral species limits the scope of the current findings and that such studies are critical for determining whether Et₃N could serve as a broad-spectrum antiviral agent. Specifically, the following revisions were made:

**Research limitations** (page 16, lines 461–466):

“Finally, although this study provides compelling in vitro evidence for the antiviral activity of Et₃N against influenza A virus, its effects on other respiratory viruses—such as respiratory syncytial virus, coronaviruses (including SARS-CoV-2), rhinoviruses, adenoviruses, and parainfluenza viruses—remain unknown. Evaluating its antiviral activity against a broader spectrum of respiratory pathogens will be essential to determine the potential of Et₃N as a broad-spectrum antiviral agent.”

**Conclusion** (page 16, lines 474–477):

“Additionally, the absence of comparative analysis with existing antivirals and the exclusive use of MDCK cells highlights the need for future investigations employing human-derived cell lines and broader respiratory viral panels.”

**Comment 5:**

*5. Lack of comparison with standard antivirals limits understanding of its relative efficacy.*

**Response 5:**

We thank the reviewer for this insightful comment. We acknowledge that the present study did not include a direct comparison between Et₃N and standard antiviral agents such as oseltamivir or baloxavir. Our primary objective was not to propose Et₃N as a therapeutic alternative to current antivirals, but rather to explore its previously unrecognized antiviral activity and provide preliminary mechanistic insights. Although Et₃N demonstrated *in vitro* antiviral effects against influenza A virus, the IC₅₀ values observed (ranging from 178 to >1,000 µM) are significantly higher than those reported for approved antivirals. This indicates that Et₃N is considerably less potent under the tested conditions. However, these findings may point to a non-classical or indirect mechanism of action, which could serve as a basis for future structural optimization and mechanistic investigation. In response to this comment, we have added the following statements to the revised manuscript:

**Research limitations** (pages 15-16, lines 445–451):

“This study did not include a direct comparison with standard antiviral drugs such as oseltamivir or baloxavir. Although Et₃N exhibited antiviral activity against influenza A virus *in vitro*, the observed IC₅₀ values (178–>1,000 µM) are markedly higher than those of clinically approved antivirals. These relatively high IC₅₀ values indicate that Et₃N is considerably less potent under the tested conditions. However, the aim of this study was not to present Et₃N as a substitute for existing antiviral agents, but rather to explore its previously unrecognized antiviral activity. This may suggest a novel mechanism of action and serve as a foundation for future compound optimization.”

**Conclusion** (page 16, lines 475):

“Additionally, the absence of comparative analysis with existing antivirals and…”

**Comment 6:**

*So, it is highly recommended to have a paragraph to discuss the major limitations of the present study before the section of the Conclusion.*

**Response 6:**

We thank the reviewer for this important recommendation. In response, we have added a new section titled “Research limitations” before the Conclusion section. This section systematically outlines the key limitations of our study, including the lack of *in vivo* validation, the relatively high IC₅₀ values of Et₃N, the absence of comparison with standard antivirals, the unidentified molecular targets, and the use of only one cell type (MDCK cells). We also addressed the need to evaluate Et₃N’s antiviral activity against other respiratory viruses to assess its potential as a broad-spectrum agent. Additionally, we have revised the Conclusion section to ensure consistency and to reflect these limitations and future research directions. The newly added Research limitations section reads as follows:

**Research limitations** (pages 15–16, lines 431–466):

“This study demonstrated that Et₃N enhances the survival of influenza A virus-infected MDCK cells and inhibits viral infection and growth *in vitro* via mechanisms independent of viral NA and RdRp. However, several limitations should be acknowledged.

All experiments were conducted exclusively *in vitro* using MDCK cells, and the antiviral efficacy of Et₃N *in vivo* remains unconfirmed. Although the SI values of Et₃N against influenza A virus ranged from approximately 10 to over 50, its safety profile, toxicity, bioavailability and pharmacokinetic properties have not yet been evaluated in animal models. Furthermore, as shown in Table 1, the IC₅₀ values of Et₃N ranged from approximately 178 to over 1,000 µM *in vitro*, which are relatively high and may not be achievable or safe at pharmacologically relevant concentrations *in vivo*. Akesson et al. reported that the plasma half-life of Et₃N in humans is nearly three hours, suggesting that its bioavailability is not negligible [14]. Therefore, further structural optimization would be required to improve its antiviral potency and reduce cytotoxicity for potential *in vivo* applications.

This study did not include a direct comparison with standard antiviral drugs such as oseltamivir or baloxavir. Although Et₃N exhibited antiviral activity against influenza A virus *in vitro*, the observed IC₅₀ values (178–>1,000 µM) are markedly higher than those of clinically approved antivirals. These relatively high IC₅₀ values indicate that Et₃N is considerably less potent under the tested conditions. However, the aim of this study was not to present Et₃N as a substitute for existing antiviral agents, but rather to explore its previously unrecognized antiviral activity. This may suggest a novel mechanism of action and serve as a foundation for future compound optimization.

The molecular mechanisms underlying the antiviral activity of Et₃N remain unclear. While Et₃N did not inhibit NA or RdRp activity, its precise targets—whether viral components or host cellular factors—are still unknown. Further mechanistic studies are necessary to determine whether Et₃N interferes with viral entry, intracellular trafficking, host signaling pathways, or membrane dynamics involved in viral replication and release.

The antiviral effects of Et₃N were demonstrated only in MDCK cells. The reproducibility of these findings in human-derived systems, such as primary bronchial epithelial cells or lung organoids, has not yet been validated. Such studies would provide more physiologically relevant insights into the potential therapeutic application of Et₃N in humans.

Finally, although this study provides compelling in vitro evidence for the antiviral activity of Et₃N against influenza A virus, its effects on other respiratory viruses—such as respiratory syncytial virus, coronaviruses (including SARS-CoV-2), rhinoviruses, adenoviruses, and parainfluenza viruses—remain unknown. Evaluating its antiviral activity against a broader spectrum of respiratory pathogens will be essential to determine the potential of Et₃N as a broad-spectrum antiviral agent.”

**Conclusion** (pages 16–17, lines 468–479):

“This study demonstrates that Et₃N enhances the survival of infected MDCK cells, and inhibits the infection and replication of influenza A H1N1 and H3N2 viruses *in vitro*. Notably, Et₃N exerts its antiviral effects through mechanisms independent of NA and RdRp, suggesting involvement of alternative viral or host cellular targets. Although the observed antiviral activity of Et₃N provides novel insights, its relatively high IC₅₀ values and lack of *in vivo* data limit its immediate therapeutic potential. Further structural optimization will be essential to improve antiviral potency and reduce cytotoxicity. Additionally, the absence of comparative analysis with existing antivirals and the exclusive use of MDCK cells highlights the need for future investigations employing human-derived cell lines and broader respiratory viral panels. Overall, our findings suggest that Et₃N serves as a novel chemical scaffold with anti-influenza potential, laying the groundwork for future development of antiviral agents that act through non-classical mechanisms.”

**Minor comments:**

**Comment 1:**
*1. No line numbers.*
**Response 1:**

We thank the reviewer for pointing this out. Line numbers have been added throughout the revised manuscript to improve readability and facilitate review.

**Comment 2:**

*2. In 2nd paragraph in the methods for MDCK cells, please mention how much you add from pen&strep mix to the cells and the same shoud be doe for the following paragrapg with P/S*
**Response 2:**

We appreciate this suggestion. We have specified the concentrations of penicillin and streptomycin in the **Materials and Methods** section as follows:

Page 4, lines 106–107:

“…, and 100 unit/mL penicillin and 100 µg/mL streptomycin…”

Page 5, line 115:

“…, 100 unit/mL penicillin and 100 µg/mL streptomycin…”

**Comment 3:**

*3. Please add key words at the end of abstract.*
**Response 3:**

Thank you for the suggestion. We have added the following keywords at the end of the **Abstract** section:

Page 2, lines 49–50:

“**Keywords**: Triethylamine, influenza virus, anti-influenza activity, infection and growth, non-toxic concentrations”

**Comment 4:**

*4. In the title of figure 2 legend, please define IF and replace it with immunoflourscence for clarity.*
**Response 4:**

We agree with this point and have revised the figure legend accordingly:

Page 10, line 266:

“**Fig 2.** **Immunofluorescence analysis of the inhibitory effect of Et_3_N on influenza A virus**…”

**Comment 5:**

*5. The title is so limited and doesn’t express the goal of your study, so please use a representative title explianing your study brifley in a few words.*

**Response 5:**

We appreciate the reviewer’s input. We have revised the title to better reflect the main findings and scope of the study:

Page 1, lines1–3:

“**Triethylamine inhibits influenza A virus infection and growth via mechanisms independent of viral neuraminidase and RNA-dependent RNA polymerase**”
